# Correction: Characterization of Subtle Brain Abnormalities in a Mouse Model of Hedgehog Pathway Antagonist-Induced Cleft Lip and Palate

**DOI:** 10.1371/journal.pone.0106607

**Published:** 2014-08-25

**Authors:** The PLOS ONE Staff

## Notice of Republication

This article was republished on July 28, 2014, due to correct the figure sizing. The publisher apologizes for this error. Please download this article again to view the corrected figures. The originally published, uncorrected article and the republished, corrected article are provided here for reference.

## Supporting Information

File S1 Originally published, uncorrected article.
(PDF)

File S2 Republished, corrected article.
(PDF)
