# Supplementary figures and images for: Characterization of Subtle Brain Abnormalities in a Mouse Model of Hedgehog Pathway Antagonist-Induced Cleft Lip and Palate
Source: PLoS One. 2014 Jul 21;9(7):e102603. doi: 10.1371/journal.pone.0102603 (PMC4105496; doi:10.1371/journal.pone.0102603)

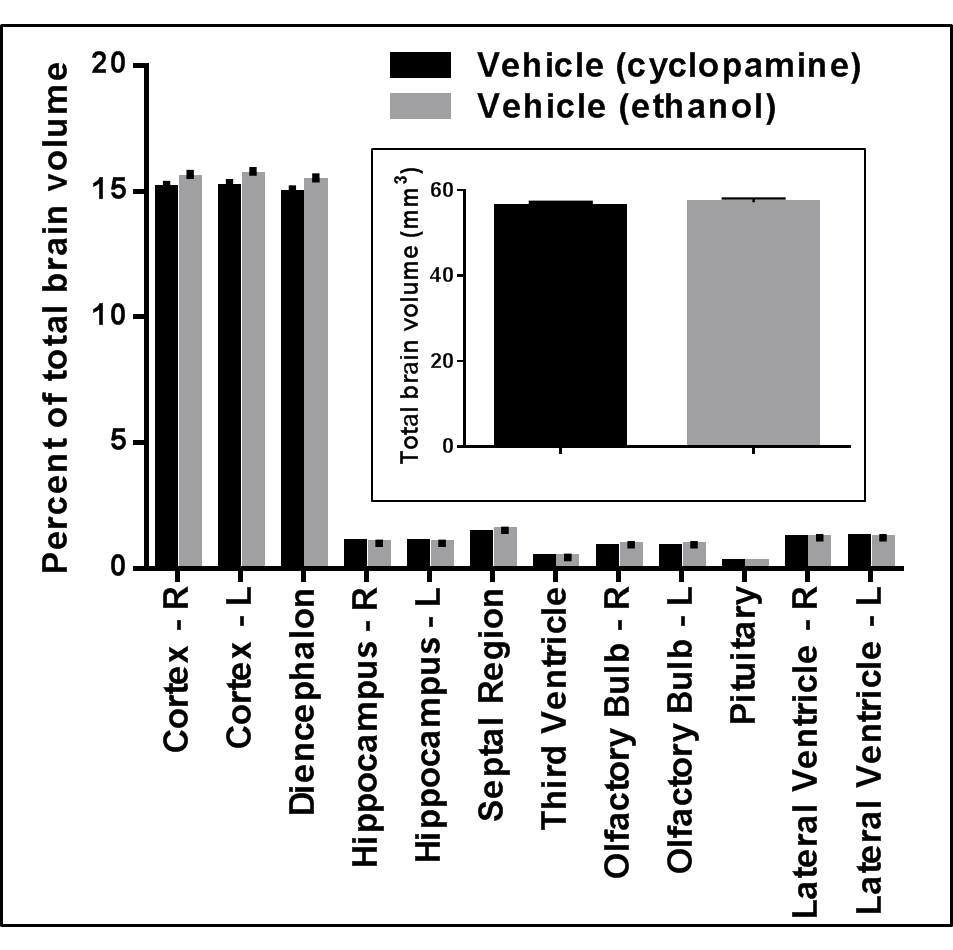

Supplement: Figure S1 — Exposure paradigm validation. The vehicle exposure group for this study [Vehicle (cyclopamine)] was compared to that of a previously examined control group exposed to lactated ringers by intraperitoneal injection at GD7 or GD8.5 [Vehicle (ethanol)]. For determination of disproportionate differences, the volume of each manually segmented brain region was calculated as a percentage of total brain volume for each animal. To illustrate relative changes on the same scale, percent volumes are normalized to mean control values. Values represent the mean ± the S.E.M. Neither total brain volume (inset) nor percent regional brain volume was significantly different between vehicle exposure groups. *p<0.05 compared to control group. (TIF) [file pone.0102603.s001.tif]
