# Supplementary material for: Correction: Characterization of Subtle Brain Abnormalities in a Mouse Model of Hedgehog Pathway Antagonist-Induced Cleft Lip and Palate
Source: PLoS One. 2014 Aug 25;9(8):e106607. doi: 10.1371/journal.pone.0106607 (PMC4143314; doi:10.1371/journal.pone.0106607)
Supplement: File S1 — Originally published, uncorrected article. (PDF) [file pone.0106607.s001.pdf]

Characterization of Subtle Brain Abnormalities in a Mouse Model of Hedgehog Pathway Antagonist-Induced Cleft Lip and Palate

Robert J. Lipinski[1,2]*, Hunter T. Holloway[1], Shonagh K. O'Leary-Moore[1,3], Jacob J. Ament[1], Stephen J. Pecevich[1], Gary P. Cofer[4], Francois Budin[3], Joshua L. Everson[2], G. Allan Johnson[4], Kathleen K. Sulik[1,5]

1 The Bowles Center for Alcohol Studies, University of North Carolina, Chapel Hill, North Carolina, United States of America, **2** Department of Comparative Biosciences, University of Wisconsin-Madison, Madison, Wisconsin, United States of America, **3** Department of Psychiatry, University of North Carolina, Chapel Hill, North Carolina, United States of America, **4** Center for In Vivo Microscopy, Duke University, Durham, North Carolina, United States of America, **5** Department of Cell Biology and Physiology, University of North Carolina, Chapel Hill, North Carolina, United States of America

Abstract

Subtle behavioral and cognitive deficits have been documented in patient cohorts with orofacial clefts (OFCs). Recent neuroimaging studies argue that these traits are associated with structural brain abnormalities but have been limited to adolescent and adult populations where brain plasticity during infancy and childhood may be a confounding factor. Here, we employed high resolution magnetic resonance microscopy to examine primary brain morphology in a mouse model of OFCs. Transient *in utero* exposure to the Hedgehog (Hh) signaling pathway antagonist cyclopamine resulted in a spectrum of facial dysmorphology, including unilateral and bilateral cleft lip and palate, cleft of the secondary palate only, and a non-cleft phenotype marked by midfacial hypoplasia. Relative to controls, cyclopamine-exposed fetuses exhibited volumetric differences in several brain regions, including hypoplasia of the pituitary gland and olfactory bulbs, hyperplasia of the forebrain septal region, and expansion of the third ventricle. However, in affected fetuses the corpus callosum was intact and normal division of the forebrain was observed. This argues that temporally-specific Hh signaling perturbation can result in typical appearing OFCs in the absence of holoprosencephaly—a condition classically associated with Hh pathway inhibition and frequently co-occurring with OFCs. Supporting the premise that some forms of OFCs co-occur with subtle brain malformations, these results provide a possible ontological basis for traits identified in clinical populations. They also argue in favor of future investigations into genetic and/or environmental modulation of the Hh pathway in the etiopathogenesis of orofacial clefting.

Citation: Lipinski RJ, Holloway HT, O'Leary-Moore SK, Ament JJ, Pecevich SJ, et al. (2014) Characterization of Subtle Brain Abnormalities in a Mouse Model of Hedgehog Pathway Antagonist-Induced Cleft Lip and Palate. PLoS ONE 9(7): e102603. doi:10.1371/journal.pone.0102603

Editor: Jingwu Xie, Indiana University School of Medicine, United States of America

Received April 14, 2014; **Accepted** June 20, 2014; **Published** July 21, 2014

Copyright: © 2014 Lipinski et al. This is an open-access article distributed under the terms of the Creative Commons Attribution License, which permits unrestricted use, distribution, and reproduction in any medium, provided the original author and source are credited.

Data Availability: The authors confirm that all data underlying the findings are fully available without restriction. All relevant data are within the paper and its Supporting Information files.

Funding: It was funded by grant nos. U01-AA017124, U01-AA021651 and P60-AA011605 grants to KKS from the National Institute on Alcohol Abuse and Alcoholism/NIH and K99DE022101 and R00DE022101 grants to RJL from the National Institute of Dental and Craniofacial Research/NIH. The funders had no role in study design, data collection and analysis, decision to publish, or preparation of the manuscript. The content of this publication is solely the responsibility of the authors and does not necessarily represent the official views of the National Institutes of Health. This study was conducted as part of the Carolina Institute for Developmental Disabilities. MR images were acquired at the Duke Center for In Vivo Microscopy, a National Institute of Biomedical Imaging and Bioengineering (NIBIB) National Resource (8P41EB015897).

Competing Interests: The authors have declared that no competing interests exist.

* Email: rjlipinski@wisc.edu

Introduction

Clefts of the lip with or without palate (CL/P) and cleft palate only (CPO) are commonly occurring human birth defects that cause significant morbidity and require extensive medical intervention [1]. Even when comprehensive treatment is available, these malformations bring significant individual, familial, and societal burden [1,2]. The mental health of individuals born with "non-syndromic" orofacial clefts (OFCs) has been extensively studied and psychosocial impairment, particularly in relation to social interaction, has been well documented [3–5]. Cognitive impairment, with specific deficits in verbal fluency, has also been described [6–9]. While the majority function within the normal IQ range, some studies have found that affected individuals score lower than peers without clefts [7,10]. Of particular importance, the prevalence of learning disabilities in populations with OFCs has been observed to be nearly ten-fold higher than that of the general population [6,9,11–13].

Conventional wisdom has held that these behavioral and cognitive traits are secondary to the speech and hearing-related complications frequent in this population or even to social stigma related to physical appearance [14,15]. However, an alternative hypothesis has been advanced, which holds that cognitive and behavioral traits identified in populations with OFCs are, in fact, a primary problem resulting from abnormal brain development [16]. Recent neuroimaging studies support this premise with the

demonstration of consistent patterns of subtle structural brain abnormalities in adolescent and adult populations with OFCs. These studies have found that clinical populations exhibit disproportionate volume reductions of the frontal lobe, subcortical nuclei, and cerebellum [16,17]. In addition to these overall size reductions, non-uniform shifts in cerebral and cerebellar volumes have also been found [18].

Development of the face and brain is an intimately-interrelated process [19]. Along with serving as a structural scaffold, the brain provides inductive molecular signals that guide development of the adjacently developing face [20]. The Hedgehog (Hh) signaling pathway has been identified as a key molecular mediator of brain-face development. *Sonic Hedgehog* (*Shh*) is initially detected in the neuroectoderm of the ventral midbrain, with expression expanding caudally into the hindbrain, and rostrally into the diencephalon and telencephalon. Hh signaling drives medial forebrain expansion and induces ventral progenitor domains. Expression of *Shh* in the neuroectoderm of the ventral forebrain indirectly induces a parallel field of expression in the facial ectoderm [21], inducing the expression of Hh target genes, including *Gli1*, in the intervening neural crest-derived mesenchymal cell population. This forebrain signaling center is critical for growth and differentiation of the midfacial primordia [22]. *Shh* null mice exhibit profound developmental defects [23], including holoprosencephaly (HPE), a condition defined by incomplete division of the forebrain, characterized by medial forebrain deficiency, and which commonly occurs with CL/P in clinical populations [23,24]. In humans, mutations in *SHH* are the most commonly identified cause of non-chromosomal HPE, accounting for approximately 12% of such cases [25,26]. However, in a recent analysis only 36% of *SHH* mutation carriers were found to have true HPE, with the remaining carriers classified as unaffected or as having microform HPE (*i.e.* facial abnormalities in the absence of detectable neuroanatomical anomalies) [26].

When Hh signaling from the forebrain neuroectoderm is blocked, S*hh* expression is not established in the facial ectoderm. This results in attenuated growth of the frontonasal prominence, which, in the chick, causes truncation of the upper beak [21]. In the mouse, we have shown that temporally-specific exposure to the Hh pathway antagonist cyclopamine results in a deficiency of the frontonasal prominence-derived medial nasal processes, which contribute to the philtrum of the upper lip, the alveolar ridge, the primary palate, and the median nose. This manifests as clefts of the lip and palate that appear to mimic human clinical phenotypes [27].

Here, employing a refined model of cyclopamine-exposure, we set out to determine whether Hh antagonist-induced facial dysmorphology is associated with abnormal brain development. High resolution magnetic resonance microscopy (MRM) was applied for concurrent visualization and measurement of facial and brain features, while diffusion tensor imaging was used to visualize white matter fiber tracts. The findings described herein illustrate that temporally-specific inhibition of the Hh signaling pathway results in clinically-relevant facial dysmorphology in the absence of the severe brain malformations that define HPE. Importantly however, subtle volumetric differences were identified in several forebrain regions of cyclopamine-exposed fetuses, providing a potential developmental basis for characteristics previously observed in affected clinical populations. Along with human neuroimaging studies, these results support the premise that some forms of typically appearing OFCs co-occur with primary structural brain abnormalities.

Materials and Methods

Timed Mouse Mating

This study was carried out in strict accordance with the recommendations in the Guide for the Care and Use of Laboratory Animals of the National Institutes of Health. All procedures involving animals were approved by the University of North Carolina at Chapel Hill Institutional Animal Care and Use Committee (protocol number 13–081.0). C57BL/6J mice were purchased from The Jackson Laboratory. Two female mice were placed with a single male for 2 hrs in the light cycle and subsequently examined for the presence of copulation plugs, marking gestational day (GD)0.

Cyclopamine Exposure

Cyclopamine (LC Labs) was dissolved in a sodium phosphate/citrate buffer containing 30% (wt/wt) 2-hydropropyl-β-cyclodextrin and administered to timed pregnant animals by subcutaneous infusion using Alzet (Durect) microosmotic pumps as previously described [28]. Model 2001D pumps (227 µl volume capacity, dispensed at 8.4 µl/h for 27 h) were loaded with cyclopamine solution to achieve a dispensation rate of 120 mg/kg/d and surgically implanted subcutaneously at GD8.25. Control animals were administered vehicle alone following the same paradigm. To assess potential effects of the surgical procedure, pump implantation, and vehicle exposure on brain development, control animals from this study were compared to a separate control population of GD17 C57BL/6J fetuses exposed to two intraperitoneal injections of lactated Ringer's solution given four hrs apart beginning at either GD7 or GD8.5 [29]. Both control populations were examined using the same methodology. Neither total brain volume, nor regional volume percentages were significantly different between the two control groups (Fig. S1).

Magnetic Resonance Imaging

On GD 17, dams were euthanized and fetuses were removed in ice-cold phosphate-buffered saline. Following fixation in a 20:1 Bouin's fixative:Prohance (Bracco Diagnostics) solution for 9 hours, fetuses were stored at 4°C in a 200:1 solution of PBS:Prohance until imaging. Magnetic Resonance Microscopy (MRM) was performed on a 9.4 T vertical bore magnet interfaced to a GE console running Epic 12.4× (GE Medical Systems). The system is equipped with Resonance Research gradients (Resonance Research, Inc.), which achieve peak gradients of 2000 mT/m. Two fetal heads were placed in an acrylic sample holder and immersed in fomblin. 3D volume images were acquired in a 10 mm diameter ×25 mm long solenoid radiofrequency coil using a radiofrequency refocused spin echo sequence (TR = 50 ms, TE = 5.2 ms, field of view = 20×10×10 mm, matrix size = 512×256×256), resulting in isotropic spatial resolution of 39 µm. A novel acquisition strategy that amplifies the high-frequency information by selectively altering the receiver gain during the phase-encoding steps was applied to extend the dynamic range of the system, capture the higher-frequency components, and limit saturation in the central k-space [30]. Total scan time for each pair of specimens was approximately 1.1 hrs.

Brain Segmentation

Pituitary, cerebellum, and forebrain regions were manually segmented using ITK-Snap (Version 2.1.4) as previously described [29,31]. Automated skull-stripping [32] was used to generate whole brain volumes as previously described [29]. As shown in

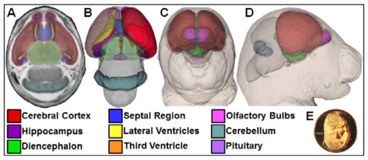

Figure 1. Magnetic resonance microscopy (MRM) enables concurrent visualization of the brain and face of GD17 mouse fetuses. Forebrain regions, pituitary, and cerebellum were manually segmented from transverse 39 μm MRM sections (A). 3D brain reconstructions were generated by overlaying manually segmented regions with whole-brain masks (B). Reduced opacity of the left cortex and diencephalon allows visualization of the left ventricle, hippocampus, third ventricle, and pituitary. From the same MRM scans, 3D head reconstructions were created, allowing concurrent visualization of the face and brain *in situ* (C–D). The size of a GD17 mouse fetus can be appreciated when shown in scale with a U.S. penny (E).
doi:10.1371/journal.pone.0102603.g001

Fig. 1, 3D reconstructions of the brain and face *in situ* were generated using Slicer3 (Version 3.6.3).

Linear Measurements

Linear measurements of snout width (SW), snout length (SL), and mandible length (ML) were obtained from 3D head reconstructions using netfabb Studio Basic (Version 4.7). For animals with UL-CLP, length measurements were taken from the side of the face with the lip cleft. Length measurements were taken from the right side for all other fetuses. Interocular distance (IOD) measurements were captured from coronal MRM images using ImageJ (Version 1.47t; http://rsb.info.nih.gov/ij/). Volumetric brain measurements were obtained with ITK-Snap (Version 2.1.4).

Diffusion Tensor Imaging

On GD19, dams were euthanized and fetuses were dissected from uterine and decidual tissue in ice-cold phosphate-buffered saline. Head regions were subsequently removed with a scalpel, fixed in a 20:1 Bouin's:Prohance solution for 9 hours, then stored at 4°C in a 200:1 solution of PBS:Prohance until imaging. Diffusion-weighted images were acquired using an RF refocused spin-echo pulse sequence (TR = 100 ms, TE = 11.8 ms, NEX = 1). The acquisition matrix was 256×256×512 over an 11×11.22 mm field of view yielding a Nyquist-limited isotropic voxel of 43 μm^3 (voxel volume = 79 pl). Diffusion preparation was accomplished using a modified Tanner–Stejskal diffusion-encoding scheme with a pair of unipolar, half-sine diffusion gradient waveforms on either side of the rf refocusing pulse. One baseline image with b = 0 (b0) and 6 high b-value images (b = 1595 s/mm^2) were acquired with diffusion sensitization along each of 6 non-collinear diffusion gradient vectors' directions [1, 1, 0], [1, 0, 1], [0, 1, 1], [−1, 1, 0], [1, 0, −1], and [0, −1, 1]. Color images were produced from raw data using DTIStudio as previously described [33].

Statistics

Multivariate analyses of variance (MANOVAs) were used to determine significant group differences in linear and volumetric measurements. Significant between-subject effects were followed by Student Newman Keuls posthoc tests when appropriate. An alpha value of 0.05 was maintained for all analyses.

Supporting Information

Validation of vehicle exposure paradigm. The control population for this study was exposed *in utero* to the cyclopamine vehicle (sodium phosphate/citrate buffer containing 30% (wt/wt) 2-hydropropyl-β-cyclodextrin) using Alzet microosmotic pumps subcutaneously implanted in timed-pregnant dams. Total brain volume and regional volume percentages from this control population were compared to those of a previously described control population exposed to a vehicle for ethanol (lactated Ringer's solution) by intraperitoneal injection at either GD7 or GD8.5 [29]. Image acquisition and processing, brain segmentation, and volumetric analysis were performed using the same methodologies for both data sets.

Gross Visualization of the Corpus Callosum. Following fixation in Bouin's solution, GD19 fetuses were hemisected in the sagittal plane with a scalpel. 20 μls of undiluted Harris Modified Hematoxylin solution was placed on the cut surface of the brain and allowed to penetrate for 20 min. Bright-field images were captured with a MicroPubisher 5.0 camera using QCapture Suite software and subsequently converted to grayscale.

Results

To examine face-brain dysmorphology, GD17 fetuses exposed to cyclopamine or vehicle alone were imaged by MRM (Fig. 1). For each fetus, facial morphology was assessed by gross inspection under a light microscope and examination of serial coronal MRM images. While vehicle-exposed control fetuses were grossly normal, facial dysmorphology was observed in each of the six cyclopamine-exposed litters. For subsequent analyses, affected fetuses were grouped by facial phenotype, including: unilateral cleft lip and palate (UL-CLP; n = 10), bilateral cleft lip and palate (BL-CLP; n = 10), cleft of the secondary palate only (CPO; n = 5), and non-cleft (NC; n = 20) (table 1). In the NC group, midfacial hypoplasia of varying severity was observed (Fig. 2). Relative to the vehicle-exposed control group, snout width was reduced in the NC and CPO groups but increased in the UL-CLP and BL-CLP groups. In each cyclopamine-exposed group, snout length and mandible length was decreased, while interocular distance was increased.

Primary brain morphology was then examined in MRM sections and 3D brain reconstructions as illustrated in Fig. 3 for representative animals from each group. Along with illustrating

Table 1. Sample size by treatment group.

Treatment group	Litters collected	Fetuses collected	Non-cleft	UL-CLP	BL-CLP	CPO
Vehicle	5	39*	39	0	0	0
Cyclopamine	6	45	20	10**	10	5

*18 fetuses were chosen at random for imaging analysis.
**n = 3 right side UL-CLP, n = 7 left side UL-CLP.
doi:10.1371/journal.pone.0102603.t001

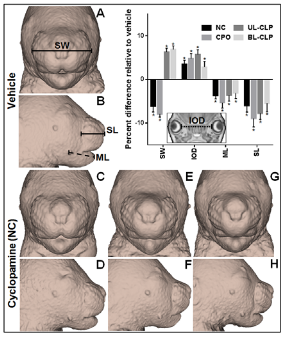

Figure 2. Cyclopamine-induced facial dysmorphology. From MRM images, extracted facial surfaces are shown for a vehicle-exposed control fetus (A,B), along with three fetuses representative of the phenotypic spectrum observed in the cyclopamine-exposed non-cleft (NC) group (C–H). Snout width (SW), snout length (SL), and mandible length (ML) were measured from facial reconstructions, while inter-ocular distance was measured from coronal MRM sections (inset). For each cyclopamine-exposed group, linear measurements are reported as percent difference relative to the vehicle exposure group. Values represent the mean + S.E.M. * $p<0.05$ compared to vehicle-exposed control group.
doi:10.1371/journal.pone.0102603.g002

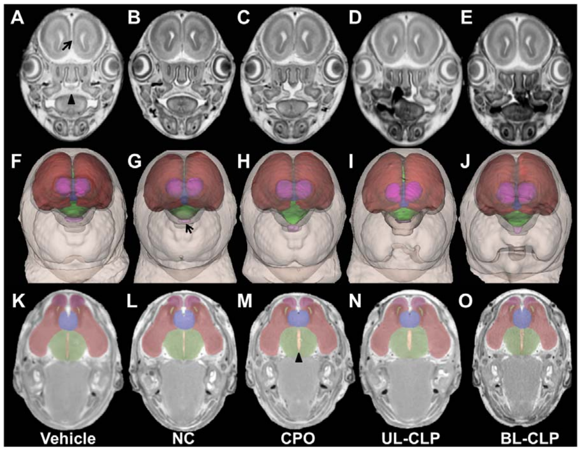

Figure 3. Cyclopamine-exposed fetuses are not holoprosencephalic. Along with a vehicle exposed control (A, F, K), representative examples of the cyclopamine-exposed NC (B, G, L), CPO (C, H, M) UL-CLP (D, I, N), BL-CLP (E, J, O), groups are shown. For each example, a coronal MRM section (A–E) showing normal separation between the cerebral hemispheres (arrow) and the secondary palate (arrow head) is shown above a reconstruction of the face and brain (F–J) and a transverse section through the forebrain (K–O). Complete separation of the cerebral hemispheres is evident in each of the reconstructed brains. Transverse sections show normal division of the cerebral cortices with an intact septal region. These images also illustrate deficiency of the pituitary (arrow in G) and olfactory bulbs, and enlargement of the third ventricle (arrow head in M) and septal region in cyclopamine-exposed fetuses. Color-coding in F-O is shown in Fig. 1, where dark red = cerebral cortices, light green = diencephalon, dark blue = septal region, yellow = lateral ventricles, orange = third ventricle, pink = olfactory bulbs, light purple = pituitary.
doi:10.1371/journal.pone.0102603.g003

normal division of the cerebral cortices in all groups, coronal sections confirmed clefts of the secondary palate in the CPO, UL-CLP, and BL-CLP groups. 3D reconstructions and transverse sections illustrate normal division of the forebrain with an intact septal region in vehicle and cyclopamine-exposed animals. However, relative to the vehicle control, several brain regions appear dysmorphic in cyclopamine-exposed fetuses. Indeed, quantitative analysis revealed that total brain volume was reduced in each of the cyclopamine-exposed groups with overt clefts, while disproportionate changes in regional brain volumes were detected in each of the cyclopamine-exposed groups (Fig. 4). Specifically, pituitary volume was reduced in each of the groups, while olfactory bulb volume was reduced in the UL-CLP and BL-CLP groups. Increased volume of the forebrain septal region was found in each of the cyclopamine-exposure groups, and volume of the third ventricle was significantly increased in groups with OFCs. These affected regions can be visualized in the transverse sections and reconstructions shown in Fig. 3.

With its complete or partial agenesis being a hallmark neuroanatomical feature of HPE [34,35], we next examined the morphology of the corpus callosum in cyclopamine-exposed fetuses with UL-CLP and BL-CLP, along with vehicle-exposed controls. Diffusion tensor imaging was applied to generate directionally encoded color maps of white matter fiber tracts in GD19 fetuses. This stage was chosen because increased myelination and size facilitated better visualization of white matter fiber tracts. In each of three fetuses with cyclopamine-induced UL-CLP that were examined, the corpus callosum, as well as the hippocampal and anterior commissures were present and not grossly hypoplastic relative to the vehicle-exposed controls (Fig. 5). This result was paralleled in three vehicle- and three cyclopamine-exposed fetuses with BL-CLP examined by gross histological staining (Fig. S2).

Discussion

Along with the findings of human neuroimaging studies and a recent examination of brain morphology in *Irf6* heterozygous adult mice [36], the results presented here support the hypothesis that some forms of OFCs co-occur with brain dysmorphology. These largely congruent observations from clinical cohorts and highly controlled animal models should prompt increased clinical awareness of primary brain abnormalities that preferentially occur in patients with OFCs. As described herein, observed abnormalities of the pituitary, olfactory bulbs, and septal region may provide an ontological basis for traits previously described in clinical populations. One such clinical study described olfaction deficits in adult males born with OFCs [37], while short stature has been well characterized and linked to possible pituitary dysfunction in affected patient populations [38–40]. While a recent study found no significant difference in pituitary volume between adult patients with OFCs and controls without clefts, pituitary structure and function were not specifically examined [41]. Consistent with increased volume of the forebrain septal region as described here, neuroimaging studies have identified shifts in tissue distribution in which the anterior cerebrum is enlarged [16,42]. Given the developmental and anatomical relationship to the septum pellucidum, the observation of septal region hyperplasia is developmentally consistent with the finding of increased incidence of cavum septum pellucidum in adult males with OFCs [43]. Enlargement of the third ventricle, as observed in cyclopamine-exposed fetuses with OFCs, has been described in human clinical populations with neurocognitive deficits, as well as in fetal mice exposed prenatally to ethanol [29,31,44,45]. While it is thought that ventricular enlargement may be indicative of cognitive dysfunction, a causative relationship has not been clearly established.

Previously, we demonstrated that the brains of mouse fetuses with cyclopamine-induced CL/P are not grossly holoprosence-

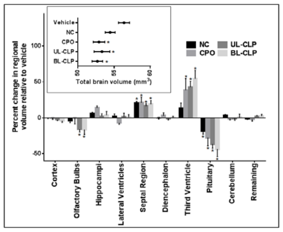

Figure 4. Volumetric brain abnormalities in cyclopamine-exposed fetuses. Total brain volumes (inset) were derived following automated skull stripping. Values represent the mean + S.E.M. * $p<0.05$ compared to the vehicle-exposed control group. For determination of disproportionate differences, the volume of each manually segmented brain region was calculated as a percentage of total brain volume for each animal. Remaining volume includes mid- and hindbrain regions. To illustrate relative changes on the same scale, percent volumes are normalized to mean control values. Values represent the mean ± the S.E.M. $^{*}p<0.05$ compared to the control group.
doi:10.1371/journal.pone.0102603.g004

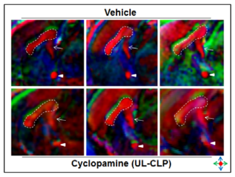

Figure 5. Corpus callosum integrity in fetuses with UL-CLP. Diffusion tensor imaging was used to visualize white matter fiber tracts in GD19 vehicle-exposed (top row) and cyclopamine-exposed fetuses with UL-CLP (bottom row). For each group, one fetus was selected at random from each of three independent litters. The directionality of the brain fiber tracts in the color-coded maps are indicated as follows: red, left/right; blue, inferior/superior; and green, anterior/posterior. In both controls and cyclopamine-exposed fetuses, the corpus callosum (dashed outline), hippocampal commissure (arrow) and anterior commissure (arrowhead) are evident.
doi:10.1371/journal.pone.0102603.g005

phalic [27]. However, the throughput and resolution of the employed methodology limited analysis to a small sample population and was incompatible with regional brain segmentation and volumetric analysis. The imaging platform used for this study enabled near-histologic resolution of anatomical sections and visualization of white matter fiber tracts. With a sample size sufficient to examine phenotypic subpopulations, we demonstrate that the applied cyclopamine exposure paradigm results in facial dysmorphism in the absence of the defining characteristics of HPE. Specifically, cyclopamine-exposed fetuses exhibited separate cerebral hemispheres, an intact corpus callosum, and a surprising increase in forebrain septal region volume. Moreover, a slight but significant increase in interocular distance was found in each of the cyclopamine-exposed groups with clefts. While HPE is typically associated with reduced interocular distance [35], clinical populations with non-syndromic OFCs have been described as hyperteloric or as having normal interocular distance [46].

We have also shown that cyclopamine-induced cleft lip results from a medial nasal process tissue deficiency [27]. This facial structure receives molecular, cellular, and structural input from the developing forebrain. While forebrain abnormalities have been described in clinical populations with CL/P, how these may relate to this initial pathogeneses of clefting is less clear because direct observation of early morphogenesis in affected human embryos is not readily done. However, several lines of evidence suggest that the morphology of the upper lateral incisor in patients with cleft lip provides important clues regarding the genesis of the clefting defect. The lateral incisor has dual origins, with two distinct dental epithelial thickenings present on the medial nasal and maxillary processes uniting following closure of the upper lip to form this tooth [47]. In individuals with cleft lip and palate, four distinct upper lateral incisor patterns occur and entail each permutation of the presence or absence of a small tooth on either side of the cleft site. One study of primary dentition in a large sample size found that a single tooth lateral to the cleft was present in 82% of cases [48]. The absence of a small lateral incisor component medial to the cleft site implies a deficiency of the medial nasal process was operational in the pathogenesis of clefting in these individuals. The apparent commonality of medial nasal process deficiency and subtle forebrain abnormalities bolsters the translational potential of the data acquired from the mouse model examined here to the clinical condition.

In addition to technological advances, this study also benefited from practical methodological refinements of a previously used cyclopamine exposure paradigm [27,28]. Specifically, timed-mating periods were reduced from overnight to two hours, allowing for increased stage specificity, and cyclopamine was purchased from LC Labs following in-house testing demonstrating increased purity and solubility of the compound compared to previously used sources. This allowed us to reduce the rate of cyclopamine infusion to 120 mg/kg/d while maintaining teratogenic efficacy. While circumventing dam toxicity, these refinements led to a marked increase in interlitter penetrance, with fetuses with clefts present in all six cyclopamine-exposed litters. In addition to previously described cleft lip and palate phenotypes, animals with clefts of the secondary palate only were also observed, albeit infrequently. While it is unclear how cyclopamine exposure causes these phenotypes that are traditionally considered disparate, a role for Hh signaling in secondary palate development and CPO has been well described in mouse models [49].

Slightly more than half of all cyclopamine-exposed fetuses did not present with clefts. However, the hypertelorism seen in animals with clefts was recapitulated in this group, as were most of the identified volumetric brain changes. Presenting with midfacial hypoplasia, this group could be considered as having a "sub-cleft" phenotype, which appears to be clinically relevant. Weinberg et al. found significant disparity between "unaffected" parents of children with overt clefts, and controls, with the former exhibiting midfacial retrusion and excess interorbital width [50]. Together, these findings argue that clefting is not a simple binary outcome and that intermediate phenotypes may provide important etiological insights.

Prevention strategies for non-syndromic CL/P and CPO are limited because our current understanding of causative factors is inadequate. The findings presented here argue that temporally-specific perturbation of the Hh signaling pathway results in a spectrum of clinically-relevant facial dysmorphology, including cleft lip and palate, cleft palate only, and a sub-cleft phenotype. While not meeting the criteria for HPE, affected animals exhibited subtle volumetric brain abnormalities that provide a possible ontological basis for traits described in clinical cohorts with non-syndromic OFCs. Taken together, these findings argue that efforts to identify genetic and environmental Hh signaling pathway modifiers could provide new insights and potential preventative strategies for these common and morbid human birth defects.

Supporting Information

Figure S1 Exposure paradigm validation. The vehicle exposure group for this study [Vehicle (cyclopamine)] was

compared to that of a previously examined control group exposed to lactated ringers by intraperitoneal injection at GD7 or GD8.5 [Vehicle (ethanol)]. For determination of disproportionate differences, the volume of each manually segmented brain region was calculated as a percentage of total brain volume for each animal. To illustrate relative changes on the same scale, percent volumes are normalized to mean control values. Values represent the mean ± the S.E.M. Neither total brain volume (inset) nor percent regional brain volume was significantly different between vehicle exposure groups. *$p<0.05$ compared to control group.
(TIF)

Figure S2 **Corpus callosum integrity in fetuses with BL-CLP.** Vehicle exposed control (top row) and cyclopamine-exposed GD19 fetuses (bottom row) were hemisected in the sagittal plane. The corpus callosum (dashed outline) was visualized using concentrated hematoxylin stain. For each group, one fetus was selected at random from each of three independent litters.
(TIF)

Author Contributions

Conceived and designed the experiments: RJL KKS GAJ. Performed the experiments: RJL HTH SKO-M GPC. Analyzed the data: RJL HTH SKO-M JJA SJP FB JLE. Contributed to the writing of the manuscript: RJL KKS GAJ.

References

1. Robin NH, Baty H, Franklin J, Guyton FC, Mann J, et al. (2006) The multidisciplinary evaluation and management of cleft lip and palate. South Med J 99: 1111–1120.
2. Waitzman N, Scheffler RM, Romano PS (1996) The cost of birth defects: estimates of the value of prevention. Lanham, Md.: University Press of America. xiv, 262 p.
3. Endriga MC, Kapp-Simon KA (1999) Psychological issues in craniofacial care: state of the art. Cleft Palate Craniofac J 36: 3–11.
4. Collett BR, Speltz ML (2007) A developmental approach to mental health for children and adolescents with orofacial clefts. Orthod Craniofac Res 10: 138–148.
5. Conrad AL, Richman L, Nopoulos P, Dailey S (2009) Neuropsychological Functioning in Children with Non-Syndromic Cleft of the Lip and/or Palate. Child Neuropsychol: 1–14.
6. Collett BR, Stott-Miller M, Kapp-Simon KA, Cunningham ML, Speltz ML (2010) Reading in children with orofacial clefts versus controls. J Pediatr Psychol 35: 199–208.
7. Nopoulos P, Berg S, VanDemark D, Richman L, Canady J, et al. (2002) Cognitive dysfunction in adult males with non-syndromic clefts of the lip and/or palate. Neuropsychologia 40: 2178–2184.
8. Jocelyn LJ, Penko MA, Rode HL (1996) Cognition, communication, and hearing in young children with cleft lip and palate and in control children: a longitudinal study. Pediatrics 97: 529–534.
9. Wehby GL, Collet B, Barron S, Romitti PA, Ansley TN, et al. (2014) Academic Achievement of Children and Adolescents With Oral Clefts. Pediatrics.
10. Richman LC (1980) Cognitive patterns and learning disabilities of cleft palate children with verbal deficits. J Speech Hear Res 23: 447–456.
11. Broder HL, Richman LC, Matheson PB (1998) Learning disability, school achievement, and grade retention among children with cleft: a two-center study. Cleft Palate Craniofac J 35: 127–131.
12. Richman LC, Eliason MJ, Lindgren SD (1988) Reading disability in children with clefts. Cleft Palate J 25: 21–25.
13. Richman LC, Ryan SM (2003) Do the reading disabilities of children with cleft fit into current models of developmental dyslexia? Cleft Palate Craniofac J 40: 154–157.
14. Estes RE, Morris HL (1970) Relationships among intelligence, speech proficiency, and hearing sensitivity in children with cleft palates. Cleft Palate J 7: 763–773.
15. Sak RJ, Ruben RJ (1982) Effects of recurrent middle ear effusion in preschool years on language and learning. J Dev Behav Pediatr 3: 7–11.
16. Nopoulos P, Berg S, Canady J, Richman L, Van Demark D, et al. (2002) Structural brain abnormalities in adult males with clefts of the lip and/or palate. Genet Med 4: 1–9.
17. Nopoulos P, Langbehn DR, Canady J, Magnotta V, Richman L (2007) Abnormal brain structure in children with isolated clefts of the lip or palate. Arch Pediatr Adolesc Med 161: 753–758.
18. Weinberg SM, Andreasen NC, Nopoulos P (2009) Three-dimensional morphometric analysis of brain shape in nonsyndromic orofacial clefting. J Anat 214: 926–936.
19. Kjaer I (1995) Human prenatal craniofacial development related to brain development under normal and pathologic conditions. Acta Odontol Scand 53: 135–143.
20. Marcucio RS, Young NM, Hu D, Hallgrimsson B (2011) Mechanisms that underlie co-variation of the brain and face. Genesis 49: 177–189.
21. Marcucio RS, Cordero DR, Hu D, Helms JA (2005) Molecular interactions coordinating the development of the forebrain and face. Dev Biol 284: 48–61.
22. Hu D, Marcucio RS (2009) A SHH-responsive signaling center in the forebrain regulates craniofacial morphogenesis via the facial ectoderm. Development 136: 107–116.
23. Chiang C, Litingtung Y, Lee E, Young KE, Corden JL, et al. (1996) Cyclopia and defective axial patterning in mice lacking Sonic hedgehog gene function. Nature 383: 407–413.
24. Richieri-Costa A, Ribeiro LA (2010) Holoprosencephaly and holoprosencephaly-like phenotypes: Review of facial and molecular findings in patients from a craniofacial hospital in Brazil. Am J Med Genet C Semin Med Genet 154C: 149–157.
25. Nanni L, Ming JE, Bocian M, Steinhaus K, Bianchi DW, et al. (1999) The mutational spectrum of the sonic hedgehog gene in holoprosencephaly: SHH mutations cause a significant proportion of autosomal dominant holoprosencephaly. Hum Mol Genet 8: 2479–2488.
26. Solomon BD, Bear KA, Wyllie A, Keaton AA, Dubourg C, et al. (2012) Genotypic and phenotypic analysis of 396 individuals with mutations in Sonic Hedgehog. J Med Genet 49: 473–479.
27. Lipinski RJ, Song C, Sulik KK, Everson JL, Gipp JJ, et al. (2010) Cleft lip and palate results from Hedgehog signaling antagonism in the mouse: Phenotypic characterization and clinical implications. Birth Defects Res A Clin Mol Teratol 88: 232–240.
28. Lipinski RJ, Hutson PR, Hannam PW, Nydza RJ, Washington IM, et al. (2008) Dose- and route-dependent teratogenicity, toxicity, and pharmacokinetic profiles of the hedgehog signaling antagonist cyclopamine in the mouse. Toxicol Sci 104: 189–197.
29. Lipinski RJ, Hammond P, O'Leary-Moore SK, Ament JJ, Pecevich SJ, et al. (2012) Ethanol-induced face-brain dysmorphology patterns are correlative and exposure-stage dependent. PLoS One 7: e43067.
30. Johnson GA, Ali-Sharief A, Badea A, Brandenburg J, Cofer G, et al. (2007) High-throughput morphologic phenotyping of the mouse brain with magnetic resonance histology. Neuroimage 37: 82–89.
31. Parnell SE, O'Leary-Moore SK, Godin EA, Dehart DB, Johnson BW, et al. (2009) Magnetic resonance microscopy defines ethanol-induced brain abnormalities in prenatal mice: effects of acute insult on gestational day 8. Alcohol Clin Exp Res 33: 1001–1011.
32. Oguz I, Lee J, Budin F, Rumple A, McMurray M, et al. (2011) Automatic Skull-stripping of Rat MRI/DTI Scans and Atlas Building. Proceedings - Society of Photo-Optical Instrumentation Engineers 7962: 7962251–7962257.
33. Jiang Y, Johnson GA (2011) Microscopic diffusion tensor atlas of the mouse brain. Neuroimage 56: 1235–1243.
34. Hahn JS, Barnes PD (2010) Neuroimaging Advances in Holoprosencephaly: Refining the Spectrum of the Midline Malformation. American Journal of Medical Genetics Part C-Seminars in Medical Genetics 154C: 120–132.
35. Cohen MM (2006) Holoprosencephaly: Clinical, anatomic, and molecular dimensions. Birth Defects Research Part a-Clinical and Molecular Teratology 76: 658–673.
36. Aerts A, Devolder I, Weinberg SM, Thedens D, Dunnwald M, et al. (2014) Haploinsufficiency of interferon regulatory factor 6 alters brain morphology in the mouse. Am J Med Genet A 164: 655–660.
37. Richman RA, Sheehe PR, McCanty T, Vespasiano M, Post EM, et al. (1988) Olfactory deficits in boys with cleft palate. Pediatrics 82: 840–844.
38. Rudman D, Davis T, Priest JH, Patterson JH, Kutner MH, et al. (1978) Prevalence of growth hormone deficiency in children with cleft lip or palate. J Pediatr 93: 378–382.
39. Laron Z, Taube E, Kaplan I (1969) Pituitary growth hormone insufficiency associated with cleft lip and palate. An embryonal developmental defect. Helv Paediatr Acta 24: 576–581.
40. Roitman A, Laron Z (1978) Hypothalamo-pituitary hormone insufficiency associated with cleft lip and palate. Arch Dis Child 53: 952–955.
41. van der Plas E, Caspell CJ, Aerts AM, Tsalikian E, Richman LC, et al. (2012) Height, BMI, and pituitary volume in individuals with and without isolated cleft lip and/or palate. Pediatric Research 71: 612–618.
42. Nopoulos P, Berg S, Canady J, Richman L, Van Demark D, et al. (2000) Abnormal brain morphology in patients with isolated cleft lip, cleft palate, or both: a preliminary analysis. Cleft Palate Craniofac J 37: 441–446.
43. Nopoulos P, Berg S, VanDemark D, Richman L, Canady J, et al. (2001) Increased incidence of a midline brain anomaly in patients with nonsyndromic clefts of the lip and/or palate. J Neuroimaging 11: 418–424.

44. Schimmel MS, Hammerman C, Bromiker R, Berger I (2006) Third ventricle enlargement among newborn infants with trisomy 21. Pediatrics 117: e928–931.
45. Muller M, Esser R, Kotter K, Voss J, Muller A, et al. (2013) Third ventricular enlargement in early stages of multiple sclerosis is a predictor of motor and neuropsychological deficits: a cross-sectional study. BMJ Open 3.
46. Anchlia S, Rao KS, Bonanthaya K, Anupama B, Nayak IV (2011) Ophthalmic considerations in cleft lip and palate patients. J Maxillofac Oral Surg 10: 14–19.
47. Hovorakova M, Lesot H, Peterkova R, Peterka M (2006) Origin of the deciduous upper lateral incisor and its clinical aspects. J Dent Res 85: 167–171.
48. Tsai TP, Huang CS, Huang CC, See LC (1998) Distribution patterns of primary and permanent dentition in children with unilateral complete cleft lip and palate. Cleft Palate Craniofac J 35: 154–160.
49. Cobourne MT, Green JB (2012) Hedgehog signalling in development of the secondary palate. Front Oral Biol 16: 52–59.
50. Weinberg SM, Naidoo SD, Bardi KM, Brandon CA, Neiswanger K, et al. (2009) Face shape of unaffected parents with cleft affected offspring: combining three-dimensional surface imaging and geometric morphometrics. Orthod Craniofac Res 12: 271–281.
